# Antioxidant therapy for patients with oral lichen planus: A systematic review and meta-analysis

**DOI:** 10.3389/fphar.2022.1030893

**Published:** 2022-11-10

**Authors:** Jie Bao, Chu Chen, Jiayu Yan, Yueqiang Wen, Jiamin Bian, Mengting Xu, Qin Liang, Qingmei He

**Affiliations:** ^1^ School of Clinical Medicine, Chengdu University of Traditional Chinese Medicine, Chengdu, China; ^2^ Department of Stomatology, Sichuan Integrated Traditional and Western Medicine Hospital, Chengdu, China; ^3^ School of Stomatology, North Sichuan Medical College, Nanchong, China; ^4^ School of Basic Medicine, Chengdu University of Traditional Chinese Medicine, Chengdu, China; ^5^ Department of Stomatology, Pengzhou Hospital of Traditional Chinese Medicine, Pengzhou, China; ^6^ Department of Neurological, Chongqing Shi Yong Chuan Hospital of Traditional Chinese Medicine, Chongqing, China

**Keywords:** antioxidant, lichen planus, oral, oral lichen planus, oral mucosal disease, meta-analysis

## Abstract

**Aims:** This study aimed to systematically review the efficacy and safety of antioxidants for patients with Oral lichen planus (OLP).

**Methods:** Databases, including PubMed, Web of Science, Cochrane Library, Embase, and Google Scholar, were searched up to 30 April 2022, for randomized controlled trials on the antioxidant therapy of OLP. The following endpoints were analyzed: pain score, clinical score, pain resolution rate, clinical resolution rate, and adverse effects.

**Results:** A total of 19 studies met the inclusion criteria, and 17 studies with 704 patients were included in the meta-analysis. The findings showed that antioxidant therapy could significantly reduce the pain score [standardized mean difference −0.72 (−1.36, −0.07), *P* = 0.03, *I^2^* = 87%, *PI^2^* < 0.00001] and clinical score [*SMD* −2.06 (−3.06, −1.06), *P* < 0.0001, *I^2^* = 94%, *PI^2^* < 0.00001] of patients with OLP and improve the pain resolution rate [risk ratio (RR) 1.15 (1.01, 1.31), *P* = 0.04, *I^2^* = 45%, *PI^2^* = 0.09] and clinical resolution rate [*RR* 1.40 (1.10, 1.78), *P* = 0.006, *I^2^* = 72%, *PI^2^* = 0.002].

**Conclusion:** The study demonstrated that antioxidant therapy was beneficial for patients with OLP, and antioxidants might be used to treat OLP.

**Systematic Review Registration:** https://clinicaltrials.gov/, identifier CRD4202233715.

## Introduction

Oral lichen planus (OLP) is a common oral mucosal disease with an incidence of 0.5%–2%, which mainly occurs in middle-aged women (Nosratzehi, 2018), and the rate of malignant transformation was 0.44% (Idrees et al., 2021a). The lesions are generally bilaterally symmetric, and the characteristic manifestations are the linear, circular, or flower-pattern lesions linked by white or gray-white small papules (Carrozzo et al., 2000; Alrashdan et al., 2016). Depending on the characteristics of local lesions, OLP can be classified into reticular type, papular type, plaque type, erosive type, atrophic type, and bullous type (Cheng et al., 2016). Previous studies demonstrated that the occurrence of OLP was associated with various factors, including immune, bacterial or viral infection, psychological factors, endocrine disturbance, and microcirculation disturbance (Alrashdan et al., 2016; Wei et al., 2018; Jung and Jang, 2022), The diagnosis of OLP is based on both clinical and histopathologic features. Sometimes a definite diagnosis can be made solely on the basis of a typical “white reticular streak lesion.” However, considering the long course of the disease, the complexity and diversity of the clinical manifestations, and the need for long-term treatment and monitoring, biopsy is necessary. In addition, inappropriate diagnosis often leads to treatment failure, histopathological confirmation of OLP is helpful before active treatment. Currently, the conventional treatment of OLP mainly includes topical corticosteroids (triamcinolone acetonide, betamethasone, etc.), calcineurin inhibitors (cyclosporine, tacrolimus or pimecrolimus), retinoids and phototherapy, of which the local application of corticosteroids has been acknowledged as the first-line drug therapy; the systemic application of corticosteroids is suitable only for patients with acute or refractory OLP (Lodi et al., 2005; Husein-ElAhmed et al., 2019).

Various previous studies have shown that the pathogenesis of OLP is associated with oxidative stress, which is mainly manifested as the imbalance between reactive oxygen species (ROS) and antioxidants (Upadhyay et al., 2010; Husain and Kumar, 2012). The key factor is the ROS generation stimulated by inflammatory infiltration composed by T cells and cytokines (Shirzad et al., 2014). ROS further induce cellular and DNA damages, and consequently induce cell apoptosis, while the apoptosis of keratinocytes is a hallmark of OLP (Sankari et al., 2015). A recent study showed that the levels of ROS-related biomarkers in saliva and serum/plasma significantly increased in patients with OLP, while the levels of antioxidant-related biomarkers reduced significantly (Wang et al., 2021). In line with these findings, increased nitric oxide (NO) and malondialdehyde (MDA) have also been found in patients with OLP and recommended as biomarkers for monitoring patients with OLP (Humberto et al., 2018; Alamir et al., 2019; Wang et al., 2021). These studies confirmed that the presence of substantial oxidative processes, increased oxidative damage biomarkers, and decreased anti-oxidative biomarkers in patients with OLP.

Antioxidants are biological and chemical compounds that inhibit or delay undesired oxidation reactions, which are either naturally produced in the human body or provided through foods, nutrients and specific antioxidant supplements (i.e., tablets, powders, concentrates). They are moreover acknowledged as “free radical scavengers” as they can inhibit and/or reduce the levels of free radicals to neutralize the adverse effects of ROS, thus achieving the aim of treating the relevant diseases induced by oxidative stress, such as aging, inflammation, diabetes, cardiovascular diseases, and cancer (Neha et al., 2019; Alkadi, 2020). A review published by Nosratzehi (2018) summarized that peroxidation products and antioxidants were potential biomarkers for predicting OLP, and antioxidants might serve as potential treatments. In addition, two more clinical studies showed that using antioxidants could significantly improve the clinical symptoms and signs of patients with OLP and reduce the levels of peroxidation biomarkers (Qataya et al., 2020; Eita et al., 2021). Therefore, this study aimed to comprehensively analyze previous findings on OLP treatment and systematically review whether antioxidant had treatment effects on patients with OLP.

## Materials and methods

### Protocol and registration

This systematic review aimed to evaluate whether antioxidants and placebo treatment, conventional treatment, and conventional auxiliary antioxidants treatment could improve the symptoms of patients with OLP and had definite treatment effects on patients with OLP. The protocol of this study was registered on PROSPERO (registration no. CRD42022337153). The study protocol abided by the PRISMA (Moher et al., 2009) statement, which could guarantee the scientificity and strictness of the study.

### Search strategy and eligibility criteria

Randomized controlled trials on treating patients with OLP using antioxidants, which were published before 30 April 2022, were searched from following databases, including PubMed, Web of Science, Cochrane Library, Embase, and Google Scholar. The search strategy was adjusted for specific databases, and no restrictions on language were applied. The keywords were as follows: oral lichen planus; lichen planus, oral; antioxidant; randomized controlled trial. During the search, various subtypes of antioxidants were also considered, such as lycopene, vitamins, and flavonoids (the detailed searching strategies and keywords are listed in Supplementary Material S1). The references of the relevant studies were also reviewed to further normalize the systematic study. All the studies were managed using EndNote20 software. After the duplicates were excluded, the titles, abstracts, and full texts of the published studies were further analyzed according to the predefined criteria.

The randomized controlled trials meeting the following criteria were included in this study: 1) patients clinically and histopathologically diagnosed with OLP, 2) Patients presented with painful oral lichen planus lesions, and 3) trials comparing the treatments using antioxidants versus placebo or conventional treatment versus auxiliary antioxidants treatment. The exclusion criteria were as follows: 1) lesions showing dysplasia, candidiasis and oral lichenoid lesions, 2) patients who underwent corticosteroids or other immunosuppressive treatment, 3) studies only comparing the antioxidant treatment versus conventional treatment, 4) animal studies or *in vitro* studies, and 5) retracted studies, reviews, meta-analyses, case reports, letters, personal comments, chapters of books, or raw data not suitable for statistical analysis.

### Types of outcome measures

The clinical efficacy and the safety of antioxidants for treating OLP were evaluated using the following indicators: Primary outcomes: Pain score as assessed by patient (measured at the end of the treatment course). Secondary outcomes: 1) Pain resolution in terms of changes in the pain extension as assessed by patient (measured at the end of the treatment course); 2) Clinical response (score and resolution of the disease) in terms of changes in the extension and severity (degree of erosion, erythema and reticulation) as assessed by clinicians (measured at the end of the treatment course); 3) Adverse effects, including clinical candidiasis and/or other toxic and side effects (measured at any time point).

The pain score was measured using the visual analogue scale (VAS), which ranged from 0 to 100 mm or 0–10 cm, with the lower scores indicating a lower level of pain. The clinical score was measured using the Thongprasom clinical score scale or its modification, REU score, or oral mucositis index. Pain resolution and clinical resolution were defined as transition to lower VAS score and clinical score (Thongprasom clinical score scale or its modification, REU score, or oral mucositis index.), respectively, used to indicate changes in pain scores and regression of clinical lesions. Pain resolution and clinical resolution were calculated by the following formula: [(initial score-final score)/initial score] × 100, improvement and worsening were defined as >0% and ≤0%, respectively. The treatment cycles ranged from 1 week to 6 months. The adverse effects were assessed during the treatments.

### Data extraction and quality assessment

After the search was completed, two investigators reviewed the titles, abstracts, and full texts independently to identify the published studies eligible for inclusion in this study. Published studies with disputes were solved by discussion or consulting with a third investigator.

One investigator extracted the data from all studies meeting the eligible criteria, and a second investigator independently verified the extracted data. All disagreements were solved by discussion. For studies with no available data, the authors were contacted to provide the original data. If the authors did not respond, the study was excluded. A data extraction form was designed for extracting the study characteristics and outcome. The following data were extracted from each eligible study: first author, year of publication, country, type of OLP, sample size, sex, age, intervention, outcome, and duration.

The Cochrane “risk-of-bias” tool (Supplementary Material S2) was used for evaluating the included studies. The risk of bias included seven parts: random sequence generation, allocation concealment, blinding of participants, blinding of outcome assessor, incomplete outcome data, selective reporting, and other bias. The risk of bias in each part was evaluated for all the included studies, based on which the included studies were classified into studies with low, high, and unclear risk of overall bias.

### Statistical analysis

All the indicators were subjected to meta-analysis according to the pre-planned subgroups to identify the potential sources of heterogeneities. As the interventions were different in studies, a subgroup analysis was performed to compare antioxidants versus placebo and conventional treatments versus conventional treatments plus antioxidants.

Review Manager 5.4 software was used for analyzing the extracted data. Risk ratio (RR) and corresponding 95% confidence interval (CI) were calculated for dichotomous data. The continuous data were reported using different scales, and the standardized mean difference (SMD) and 95% CI were calculated. The heterogeneity was evaluated using the I2 index, which was classified according to the Cochrane Handbook as follows: 0%–40% indicated possibly not important, 30%–60% indicated moderate heterogeneity, 50%–90% indicated substantial heterogeneity, and 75%–100% indicated considerable heterogeneity. The sensitivity analysis was performed when the heterogeneity was large or considerable to assess and verify the influences of studies on pooled analysis results.

### Level of evidence

The GRADE scoring standard (Guyatt et al., 2008; Balshem et al., 2011) was used for evaluating the quality of evidence. The evidence provided by randomized controlled trials was initially classified as high-quality evidence, which could be downgraded by the presence of the following factors: imprecision, inconsistency, indirectness, and publication bias. The analyses were stratified into two subgroups according to treatments as follows: 1) comparison of antioxidants treatment versus placebo treatment and 2) comparison of conventional treatment plus antioxidants versus conventional treatment.

## Results

### Characteristics of the included studies

**FIGURE 1:**
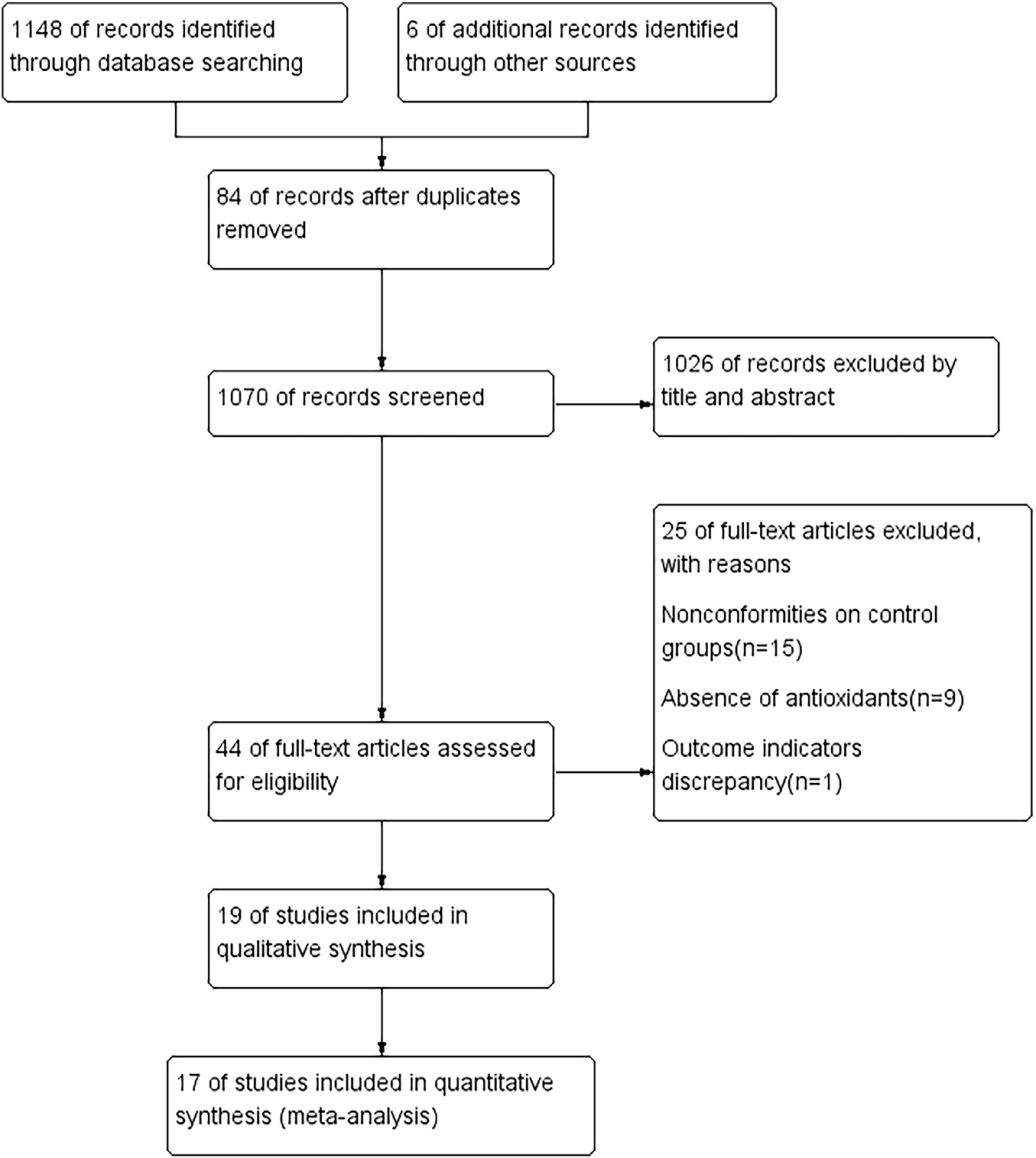
Flow diagram of literature search according to the PRISMA statement.

A total of 1154 studies were retrieved according to the search strategy. Of these, 84 repetitive studies were excluded after the studies were reviewed one by one. For the other 1070 studies, the titles and abstracts were read to exclude reviews, nonrandomized controlled trials, and randomized controlled trials with the study design not meeting the inclusion criteria. Thus, 1026 studies were excluded, and 44 studies were retrieved after the initial screening. The full texts of the 44 studies were read for secondary screening, and 15 studies with inappropriate controls, 9 studies no using antioxidants, and 1 study reporting only other endpoints were excluded; 19 studies were considered meeting the inclusion criteria (Veneri et al., 2020; Shoukheba and Elgendy, 2016; Shetty et al., 2016; Sanatkhani et al., 2014; Salazar-Sanchez et al., 2010; Saawarn et al., 2011; Nolan et al., 2009; Mostafa and Zakaria, 2018; Choonhakarn et al., 2008; Chainani-Wu et al., 2007; Chainani-Wu et al., 2012; Belal, 2015; Bakhshi et al., 2020; Bacci et al., 2017; Amirchaghmaghi et al., 2016; Agha-Hosseini et al., 2010; Agha-Hosseini et al., 2021; Abdeldayem et al., 2020; NCT02329600, 2014). Finally, 17 studies (Veneri et al., 2020; Shoukheba and Elgendy, 2016; Shetty et al., 2016; Sanatkhani et al., 2014; Salazar-Sanchez et al., 2010; Saawarn et al., 2011; Nolan et al., 2009; Mostafa and Zakaria, 2018; Choonhakarn et al., 2008; Chainani-Wu et al., 2007; Chainani-Wu et al., 2012; Bakhshi et al., 2020; Amirchaghmaghi et al., 2016; Agha-Hosseini et al., 2010; Agha-Hosseini et al., 2021; Abdeldayem et al., 2020; NCT02329600, 2014) were included in the meta-analysis. The processes of study screening and selection are shown in Figure 1.

The major characteristics of the included studies are shown in Table 1.

**TABLE 1:** Summary of the included studies.

Study	Study design	Country and participants	Sample description sample size (male/female); age (mean ± SD or median (minimum — maximum)]; case control		Intervention case control		Outcome and duration
Abdeldayem 2020	RCT	Egypt; symptomatic OLP	15 (5/10); 54.23 ± 12.79	15 (4/11); 0.82 ± 13.61	Triamcinolone acetonide four times a day and vitamin E capsule once daily in the morning	Triamcinolone acetonide four times a day and placebo capsule once daily in the morning	Pain (NRS), clinical scores (Thongprasom), and salivary total antioxidant capacity; 4 weeks
Agha-Hosseini 2010	RCT	Iran; symptomatic OLP	20 (9/11); 25–70	17 (7/10); 25–70	Purslane 235 mg, one pill per day	Placebo 235 mg, one pill per day	Pain resolution, clinical resolution, and adverse effects; 6 months
Agha-Hosseini 2021	split-mouth RCT	Iran; symptomatic OLP	27 (10/17); 49.81 ± 9.63	27 (10/17); 49.81 ± 9.63	Hyaluronic acid and triamcinolone solution injection	Triamcinolone solution injection	Pain (VAS), lesion, and adverse effects; 6 months
Amirchaghmaghi 2016	RCT	Iran; symptomatic OLP	12 (2/10); 49.42 ± 11.22	8 (3/5); 52.75 ± 9.43	Dexamethasone mouthwash 0.5 mg, Nystatin suspension three times daily, and four curcumin tablets twice daily	Dexamethasone mouthwash 0.5 mg, Nystatin suspension three times daily, and four placebo tablets twice daily	Pain (VAS), clinical score (Thongprasom), clinical efficiency, and adverse effects; 4 weeks
Bakhshi 2020	RCT	Iran; symptomatic OLP	14 (3/11); 59 ± 15.12	17 (4/13); 48 ± 12.71	Triamcinolone mouthwash and 1% nanomicelle curcumin gel, three times a day	Triamcinolone mouthwash and placebo gel, three times a day	Clinical score (REU) and clinical efficiency; 4 weeks
Chainani-Wu 2007	RCT	United States; symptomatic OLP	16 (4/12); 60.6 ± 7.5	17 (6/11); 60.6 ± 9.8	60 mg Prednisone and 2000 mg of curcuminoids 2 times a day	60 mg Prednisone and placebo two times a day	Pain resolution and adverse effects; 7 weeks
Chainani-Wu 2011	RCT	United States; symptomatic OLP	10 (2/8) 60.8 ± 8.6	10 (5/5) 56.2 ± 11.7	6,000 mg of curcuminoids 3 times a day	Identical placebo tablets three times a day	Symptom scores (NRS), clinical signs (MOMI), CRP and IL-6, adverse effects, and bleeding index; 2 weeks
Choonhakarn 2008	RCT	Thailand; OLP	27 (9/18); 52.81 ± 12.16	27 (11/16); 52.44 ± 14.85	Aloe vera gel twice a day	Placebo twice a day	Pain resolution, clinical score (Thongprasom), and clinical resolution; 8 weeks
Ghada Nabil 2016	3-arm-RCT	Egypt; symptomatic OLP	15 (6/9); 41.4 ± 9.7	15 (5/10); 44.3 ± 16.2	Triamcinolone acetonide applied topically four times a day and one green tea tablet per day	Triamcinolone acetonide applied topically four times a day	Pain (VAS), salivary total oxidative capacity, and adverse effects; 1 month
Mostafa 2018	3-arm-RCT	Egypt; symptomatic OLP	22 (9/13); 54.4 ± 4.2	22 (9/13); 56.2 ± 5.5	Ozone (60% strength) applied orally for 1 min, twice a week; triamcinolone acetonide 0.1%, four times per day	Triamcinolone acetonide 0.1%, four times per day	Pain (0–4), clinical scores (Thongprasom), and clinical resolution; 4 weeks
Nolan 2009	RCT	United Kingdom; symptomatic OLP	62 (15/47); 56.46	62 (9/53); 55.3	Topical hyaluronic acid 0.2%, up to five times a day	Placebo, up to five times a day	Pain resolution, clinical score (Thongprasom), and oral function; 4 weeks
Saawarn 2011	RCT	India; symptomatic OLP	15 (7/8); 32 ± 12.9	15 (12/3); 43.46 ± 18.2	Softgel capsule lycopene 8 mg 3 times daily	Identical placebo three times daily	Burning sensation (VAS) and clinical efficiency; 2 months
Salazar-Sanchez 2010	RCT	Spain; OLP	31 (3/28); 62.19 ± 10.45	24 (1/23); 60.71 ± 12.23	Aloe vera water suspension (70%), 0.4 ml orally for 1 min, 3 times a day	Placebo suspension (70%), 0.4 ml orally for 1 min, three times a day	Pain (VAS), clinical score (Thongprasom), clinical resolution, clinical efficiency, OHIP-49, HAD, and adverse effects; 12 weeks
Sanatkhani 2014	RCT	Iran; symptomatic OLP	15 (0/15); 46.8 ± 8.9	15 (2/13); 46.53 ± 10.75	Dexamethasone mouth rinse and fluconazole capsule 100 mg daily; 20 ml of cedar honey 3 times daily	Dexamethasone mouth rinse and fluconazole capsule 100 mg daily	Pain resolution, clinical resolution, and adverse effects; 4 Weeks
Shetty 2016	RCT	India; OLP	25 (13/12); 19–75	25 (11/14); 26–70	0.2% Hyaluronic acid orabase applied three times daily	Apply topical placebo orabase three times daily	Pain resolution and clinical score (area scores); 6 weeks
Shoukheba 2016	RCT	Egypt; symptomatic OLP	15 (3/12); 47.33 ± 8.138	15 (6/9); 49.66 ± 5.61	Triamcinolone acetonide applied topically four times a day; Coenzyme Q10 30 mg capsule three times day	Triamcinolone acetonide applied topically four times a day	Pain (VAS), clinical scores (Thongprasom), clinical resolution, and adverse effects; 12 weeks
Veneri 2020	RCT	Italy; symptomatic OLP	26 (8/18); 47–83	25 (8/17); 46–81	Double-distilled water/ozone water (2:3) rinse four times, twice a week; betamethasone sodium phosphate 500 mg soluble tablets, rinse twice a day	Double-distilled water rinse four times, twice a week; betamethasone sodium phosphate 500 mg soluble tablets, rinse twice a day	Pain resolution, clinical resolution, clinical efficiency, adverse effects, and relapse rate; 3 months

### Risk of bias

**FIGURE 2:**
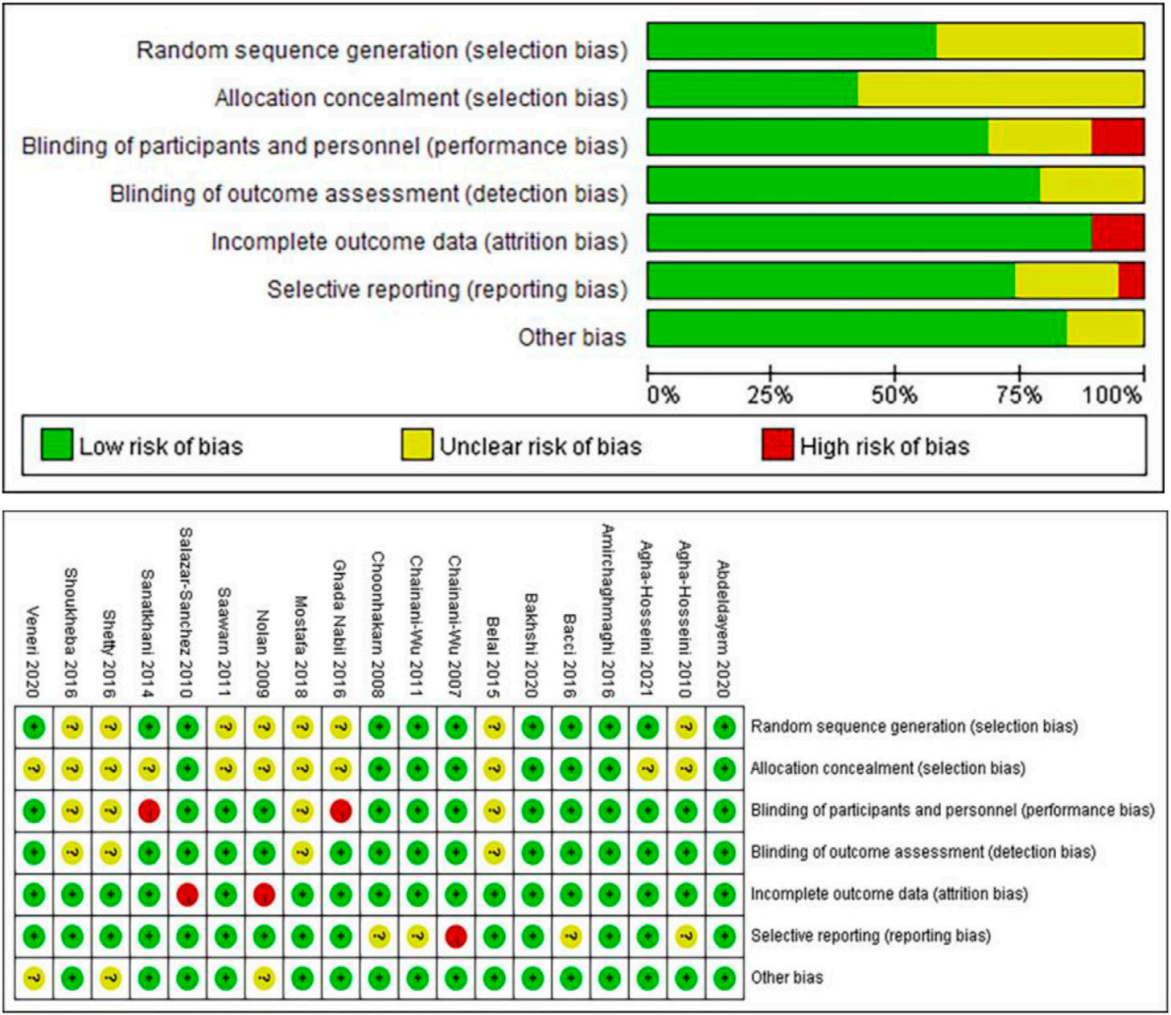
Risk of bias of the included studies (Cochrane tool).

The Cochrane tool (Higgins et al., 2011) was used to evaluate the risk of bias of the 19 randomized controlled trials. Figure 2 illustrates the risk of bias of the studies. Specifically, 3 studies (Amirchaghmaghi et al., 2016; Abdeldayem et al., 2020; Bakhshi et al., 2020) met all the criteria of bias risks and were classified with a low risk of bias; 11 studies (Choonhakarn et al., 2008; Agha-Hosseini et al., 2010; Saawarn et al., 2011; Chainani-Wu et al., 2012; Belal, 2015; Shetty et al., 2016; Shoukheba and Elgendy, 2016; Bacci et al., 2017; Mostafa and Zakaria, 2018; Veneri et al., 2020; Agha-Hosseini et al., 2021) had 1 or more items considered unclear and were classified with unclear overall risk of bias; and 5 studies (Sanatkhani et al., 2014; Salazar-Sanchez et al., 2010; Nolan et al., 2009; Chainani-Wu et al., 2007; NCT02329600, 2014) had 1 item considered with a significant risk of bias (no blinding, incomplete outcome data, selective reporting, or other bias) and were classified with a high risk of bias.

### Meta-analysis

A total of 17 studies were included in the meta-analysis, while 2 studies were excluded. Specifically, 1 study (Bacci et al., 2017) was a cross-control study, which was not included because no paired analysis method was used. The other study (Belal, 2015) did not report the endpoints for evaluation, and thus was not included in the meta-analysis. The meta-analysis results of the endpoints were as follows.

#### Meta-analysis for pain score

**FIGURE 3:**
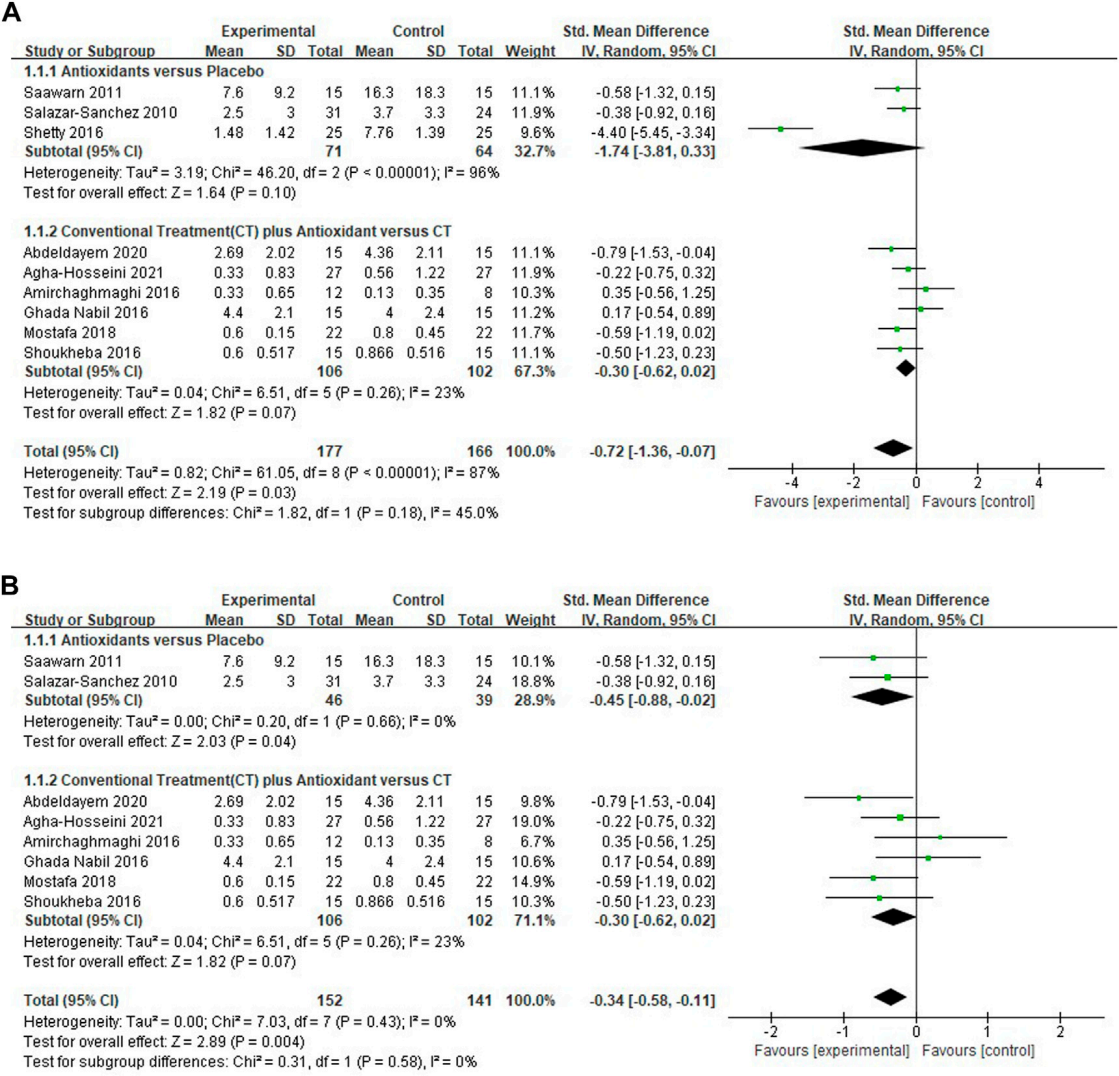
**(A)** Forest plot of pain score. **(B)** Forest plot of pain score.

Nine studies (Shoukheba and Elgendy, 2016; Shetty et al., 2016; Salazar-Sanchez et al., 2010; Saawarn et al., 2011; Mostafa and Zakaria, 2018; Amirchaghmaghi et al., 2016; Agha-Hosseini et al., 2021; Abdeldayem et al., 2020; NCT02329600, 2014) evaluating the reduction of pain scores in patients with OLP treated with antioxidants were included, the pooled analysis showed that the mean pain score was lower in the test group (*n* = 177) than in the control group (*n* = 166) [*SMD* −0.72 (−1.36 to −0.07), *p* = 0.03; Figure 3A]. The mean pain score in patients treated with antioxidants and placebos [*SMD* −1.74 (−3.81, 0.33), *p* = 0.10, *I* ^2^ = 96%, *P*_*I*_^2^ < 0.00001], conventional treatments plus antioxidants and conventional treatments [*SMD* −0.30 (−0.62 to 0.02), *p* = 0.07, *I* ^2^ = 23%, *P*_*I*_^2^ = 0.26] were similar. The overall heterogeneity of all the studies was high (*I* ^2^ = 87%, *P*_*I*_^2^ < 0.00001). The results of the subgroup analyses showed that there was no significant difference in mean pain score between subgroups stratified by different types of treatment (*p* = 0.18 > 0.05 for heterogeneity between group). The studies were excluded item by item to conduct sensitivity analysis to investigate whether some studies influenced the robustness of the results. As shown in the Table 2, sensitivity analysis suggested that the study performed by Sheety et al. (Shetty et al., 2016) may have been a potential source of heterogeneity. After excluding this study, the new level of heterogeneity becomes 0%, and the pooled mean pain score in the remaining 8 studies was −0.34 (95% CI, −0.58, −0.11, *I* ^2^ = 0%, *P*_*I*_^2^ = 0.43; *p* = 0.004; Figure 3B).

**TABLE 2:** Sensitivity analysis of pain scores using the method of eliminating studies one by one.

Deleted study	*I* ^2^ (%)	*p*	SMD (95% CI)
Saawarn 2011	89	<0.00001	−0.74 (−1.46, −0.02)
Salazar-Sanchez 2010	88	<0.00001	−0.77 (−1.52, −0.02)
Shetty 2016	0	0.43	−0.34 (−0.58, −0.11)
Abdeldayem 2020	88	<0.00001	−0.71 (−1.43, 0.01)
Agha-Hosseini 2021	88	<0.00001	−0.79 (−1.54, −0.05)
Amirchaghmaghi 2016	88	<0.00001	-0.84 (−1.52, −0.15)
Ghada Nabil 2016	88	<0.00001	−0.83 (−1.53, −0.13)
Mostafa 2018	89	<0.00001	−0.74 (−1.48, 0.00)
Shoukheba 2016	89	<0.00001	−0.75 (−1.47, −0.03)

#### Meta-analysis for clinical score

**FIGURE 4:**
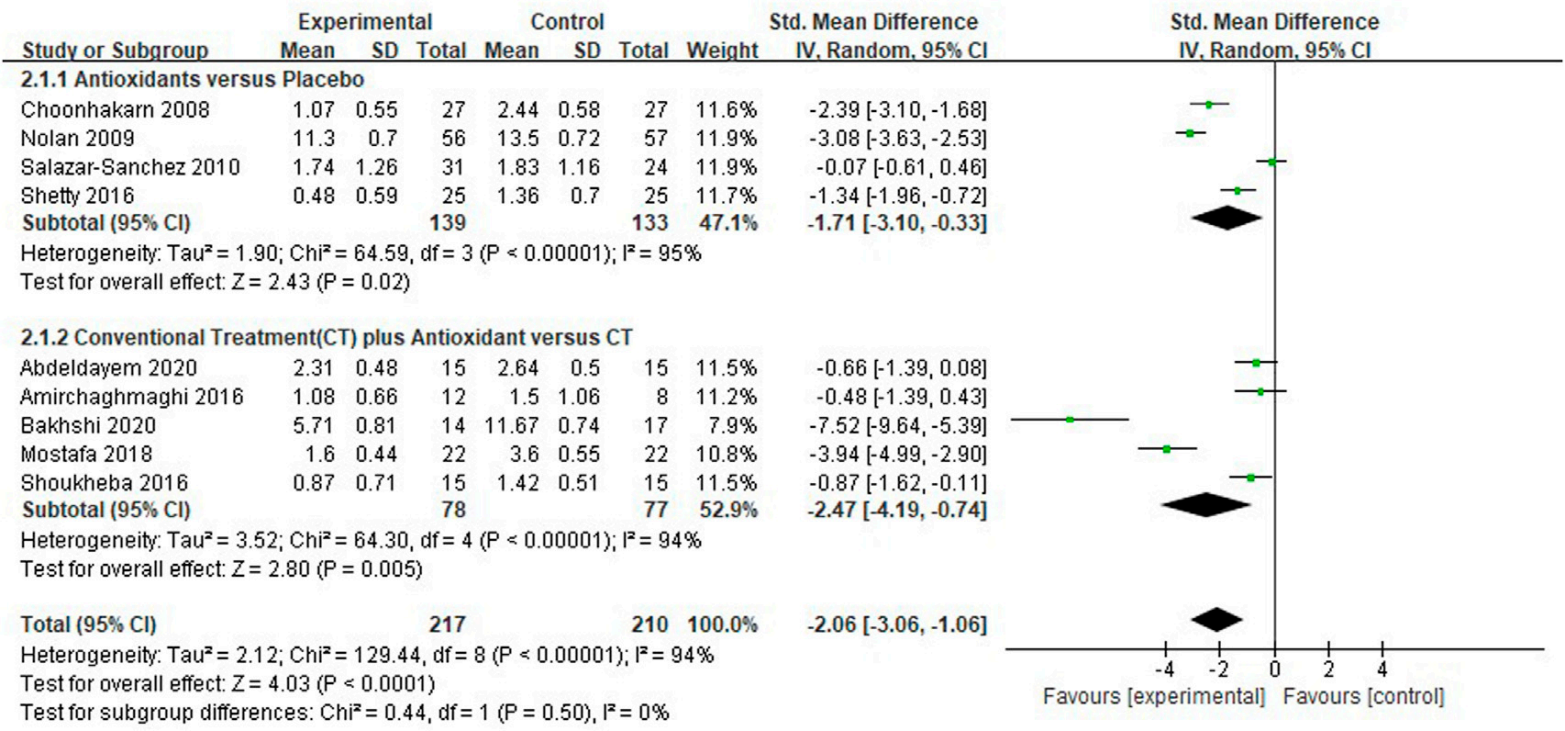
Forest plot of clinical score.

Nine studies (Choonhakarn et al., 2008; Nolan et al., 2009; Salazar-Sanchez et al., 2010; Amirchaghmaghi et al., 2016; Shetty et al., 2016; Shoukheba and Elgendy, 2016; Mostafa and Zakaria, 2018; Abdeldayem et al., 2020; Bakhshi et al., 2020) evaluated the reduction of clinical scores in patients with OLP. The mean clinical score was significantly lower in the test group compared with the control group [*SMD* −2.06 (−3.06 to −1.06), *p* < 0.0001]. The subgroup analysis showed that the mean clinical score was significantly lower in the antioxidants group than in the placebo group [*SMD* −1.71 (−3.10 to −0.33), *p* = 0.02, *I* ^2^ = 95%, *P*_*I*_^2^ < 0.0001], and significantly lower in the conventional treatment plus antioxidants group than in the conventional treatment group [*SMD* −2.47 (−4.19 to −0.74), *p* = 0.005, *I* ^2^ = 94%, *P*_*I*_^2^ < 0.0001]. The overall heterogeneity of all the studies was high (*I* ^2^ = 94%, *p* < 0.00001; Figure 4). As shown in the Table 3, during sensitivity analysis, the heterogeneity ranged from 89% to 93%, and the clinical score was not significantly influenced after each study was excluded. Also, the *I* ^2^ was not significantly changed, indicating that the results of the analysis were relatively robust.

**TABLE 3:** Sensitivity analysis of clinical scores using the method of eliminating studies one by one.

Deleted study	*I* ^2^ (%)	*p*	SMD (95% CI)
Choonhakarn 2008	92	<0.00001	−1.60 (−2.46 to −0.74)
Nolan 2009	93	<0.00001	−1.90 (−2.92 to −0.89)
Salazar-Sanchez 2010	92	<0.00001	−1.94 (−2.84 to −1.04)
Sheety 2016	93	<0.00001	−1.78 (−2.73 to −0.84)
Abdeldayem 2020	93	<0.00001	−1.86 (−2.79 to −0.94)
Amirchaghmaghi 2016	93	<0.00001	−1.87 (−2.77 to −0.97)
Bakhshi 2020	89	<0.00001	−1.25 (−1.94 to −0.56)
Mostafa 2018	90	<0.00001	−1.37 (−2.12 to −0.62)
Shoukheba 2016	93	<0.00001	−1.84 (−2.76 to −0.91)

#### Meta-analysis for pain resolution

**FIGURE 5:**
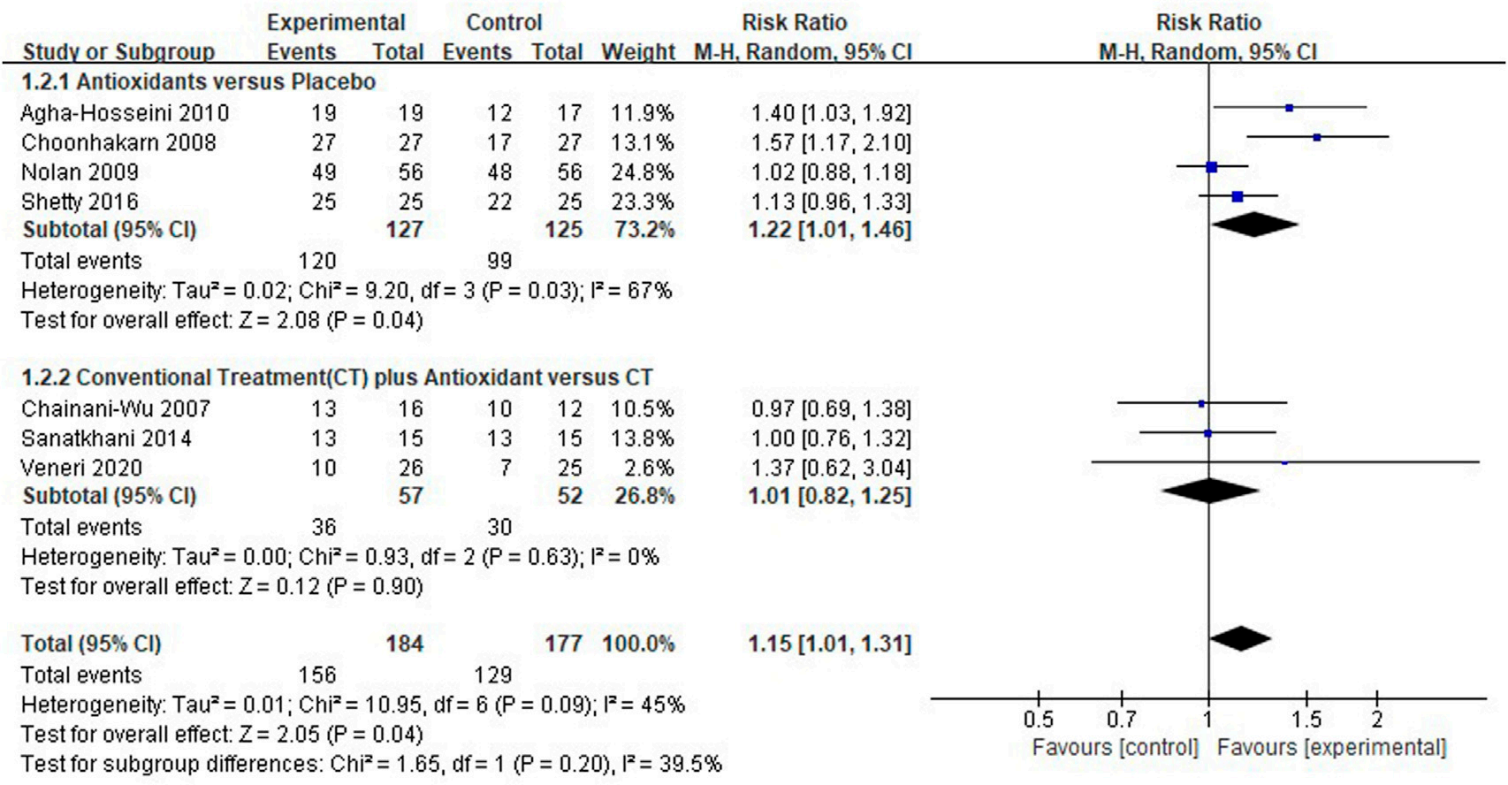
Forest plot of pain resolution.

Seven clinical studies (Chainani-Wu et al., 2007; Choonhakarn et al., 2008; Nolan et al., 2009; Agha-Hosseini et al., 2010; Sanatkhani et al., 2014; Shetty et al., 2016; Veneri et al., 2020) reported pain resolution, and the overall heterogeneity was moderate (*I* ^2^ = 45%). In the pooled results [*RR* 1.15 (1.01–1.31), *p* = 0.04, *I* ^2^ = 45%, *P*_*I*_^2^ = 0.09; Figure 5], test group (*n* = 156) presented a higher pain resolution compared to control group (*n* = 129). And respectively, the pain resolution rate was higher in patients treated with antioxidants than in patients treated only using placebo [*RR* 1.22 (1.01–1.46), *p* = 0.04, *I* ^2^ = 67%, *P*_*I*_^2^ = 0.03], the pain resolution rate was similar in patients treated with conventional treatment plus antioxidants compared to patients treated with conventional treatment only. [*RR* 1.01 (0.82–1.25), *p* = 0.90, *I* ^2^ = 0%, *P*_*I*_^2^ = 0.63].

#### Meta-analysis for clinical resolution

**FIGURE 6:**
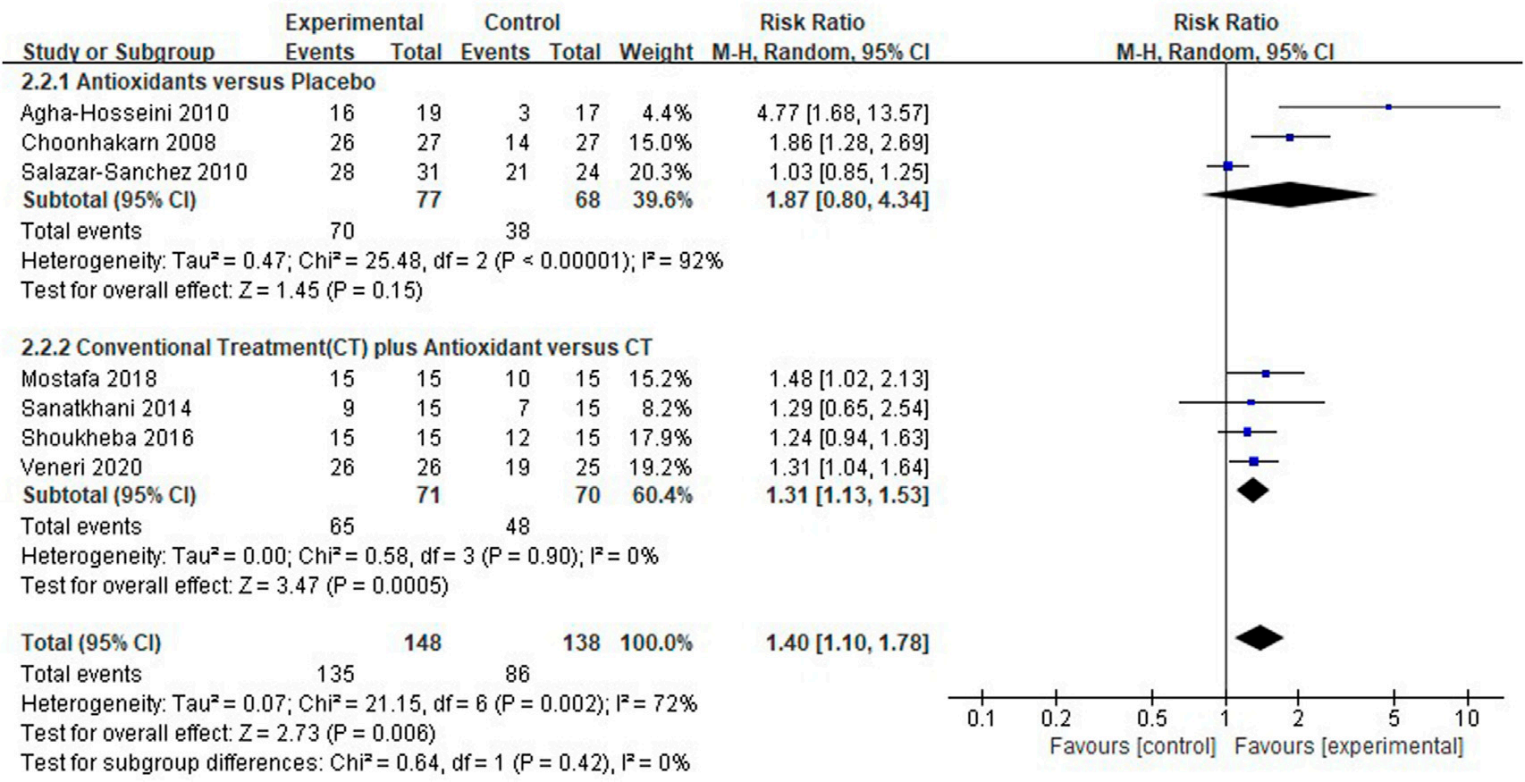
Forest plot of clinical resolution.

Seven clinical studies (Choonhakarn et al., 2008; Agha-Hosseini et al., 2010; Salazar-Sanchez et al., 2010; Sanatkhani et al., 2014; Shoukheba and Elgendy, 2016; Mostafa and Zakaria, 2018; Veneri et al., 2020) reported the clinical resolution of patients with OLP; the results showed that the use of antioxidants was still associated with the clinical resolution rate in patients with OLP [*RR* 1.40 (1.10–1.78), *p* = 0.006; Figure 6]. The antioxidants and placebo groups had similar clinical resolution rates [*RR* 1.87 (0.80–4.34), *p* = 0.15, *I* ^2^ = 92%, *P*_*I*_^2^ <0.00001], compared with the conventional treatment, the conventional treatment plus antioxidants had a higher clinical resolution rate [*RR* 1.31 (1.13–1.53), *p* = 0.0005, *I* ^2^ = 0%, *P*_*I*_^2^ = 0.90]. However, there was still high heterogeneity (*I* ^2^ = 72%, *P*_*I*_^2^ = 0.002) in clinical resolution rate, which the subgroup analysis could not explain (*p* = 0.42). During sensitivity analysis, the heterogeneity ranges from 53% to 78%.

#### Meta-analysis for adverse effects

**FIGURE 7:**
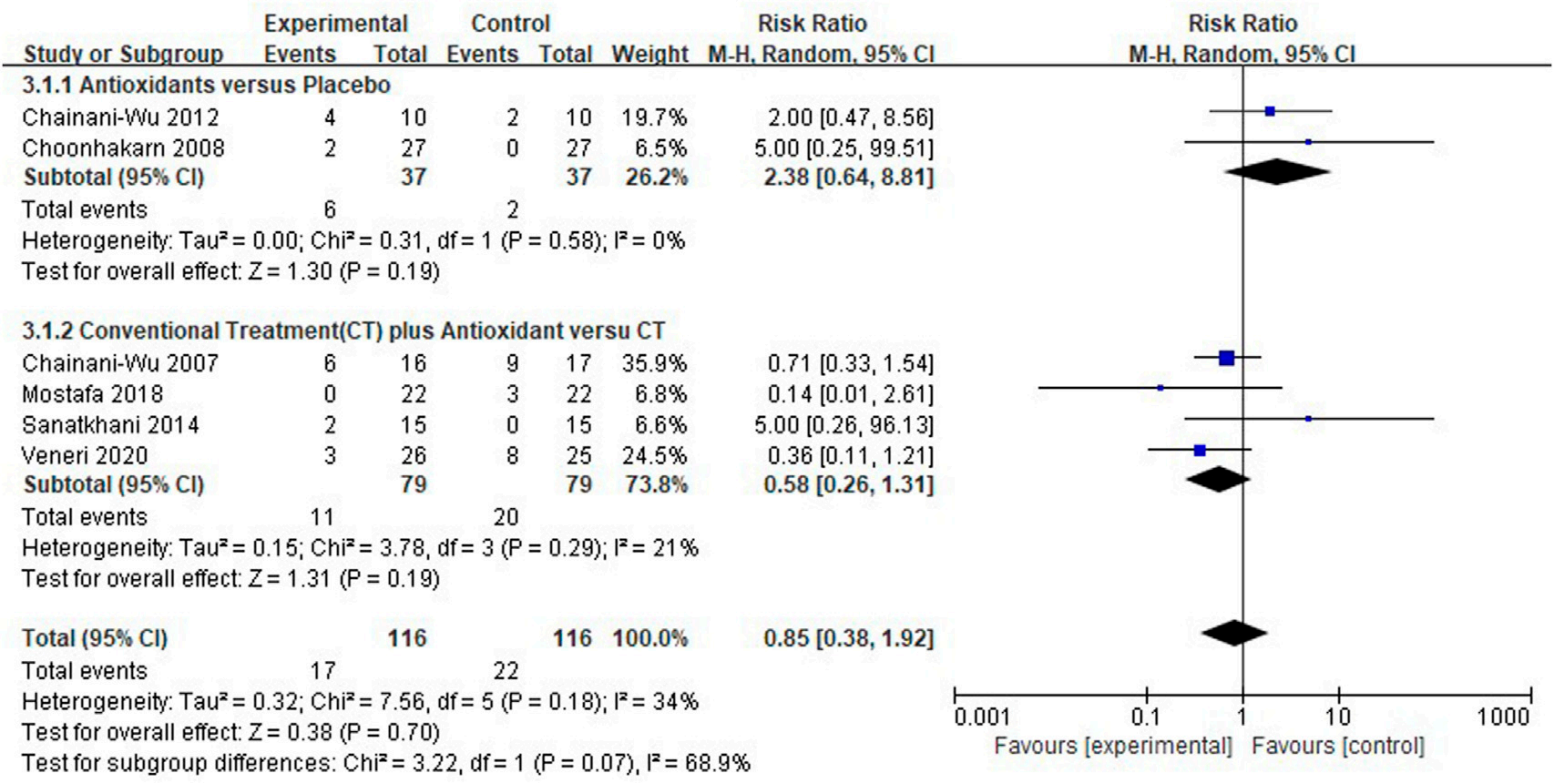
Forest plot of adverse effects.

Six clinical studies (Chainani-Wu et al., 2007; Choonhakarn et al., 2008; Chainani-Wu et al., 2012; Sanatkhani et al., 2014; Mostafa and Zakaria, 2018; Veneri et al., 2020) reported the adverse effects of antioxidant treatment, and the overall heterogeneity was relatively low (*I* ^2^ = 34%). The adverse effects were similar between the test group (*n* = 116) and the control group (*n* = 116) [*RR* 0.85 (0.38–1.92), *p* = 0.70, *I* ^2^ = 34%, *P*_*I*_^2^ = 0.18]. The comparison between antioxidants and placebo [*RR* 2.38 (0.64–8.81), *p* = 0.19, *I* ^2^ = 0%, *P*_*I*_^2^ = 0.58], and between conventional treatment plus antioxidants and conventional treatment [*RR* 0.58 (0.26–1.31), *p* = 0.19, *I* ^2^ = 21%, *P*_*I*_^2^ = 0.29; Figure 7] both showed similar results. The findings showed that the adverse effects were not significantly different between the test and control groups. Adverse events reported by the studies as shown in the Table 4.

**TABLE 4:** Adverse events reported by the studies.

Group	Study	Adverse events in the test group	Adverse events in the control group
Antioxidants versus placebo	Chainani-Wu 2012	Diarrhea; Constipation; Abdominal pain; Heartburn; Nausea	Diarrhea; Constipation
	Choonhakarn 2008	Stinging; Mild itching	
Conventional treatment (CT) plus antioxidants versus CT	Chainani-Wu2007	Headache; Rash; Flatulence; Pitted fingernails; Dry mouth	Headache; Rash; Dry mouth; Metallic taste
	Mostafa2018		Oral candidiasis
	Sanatkhani 2014	Mild burning sensation	
	Veneri2020	Oral candidiasis	Oral candidiasis

#### Level of evidence

**FIGURE 8:**
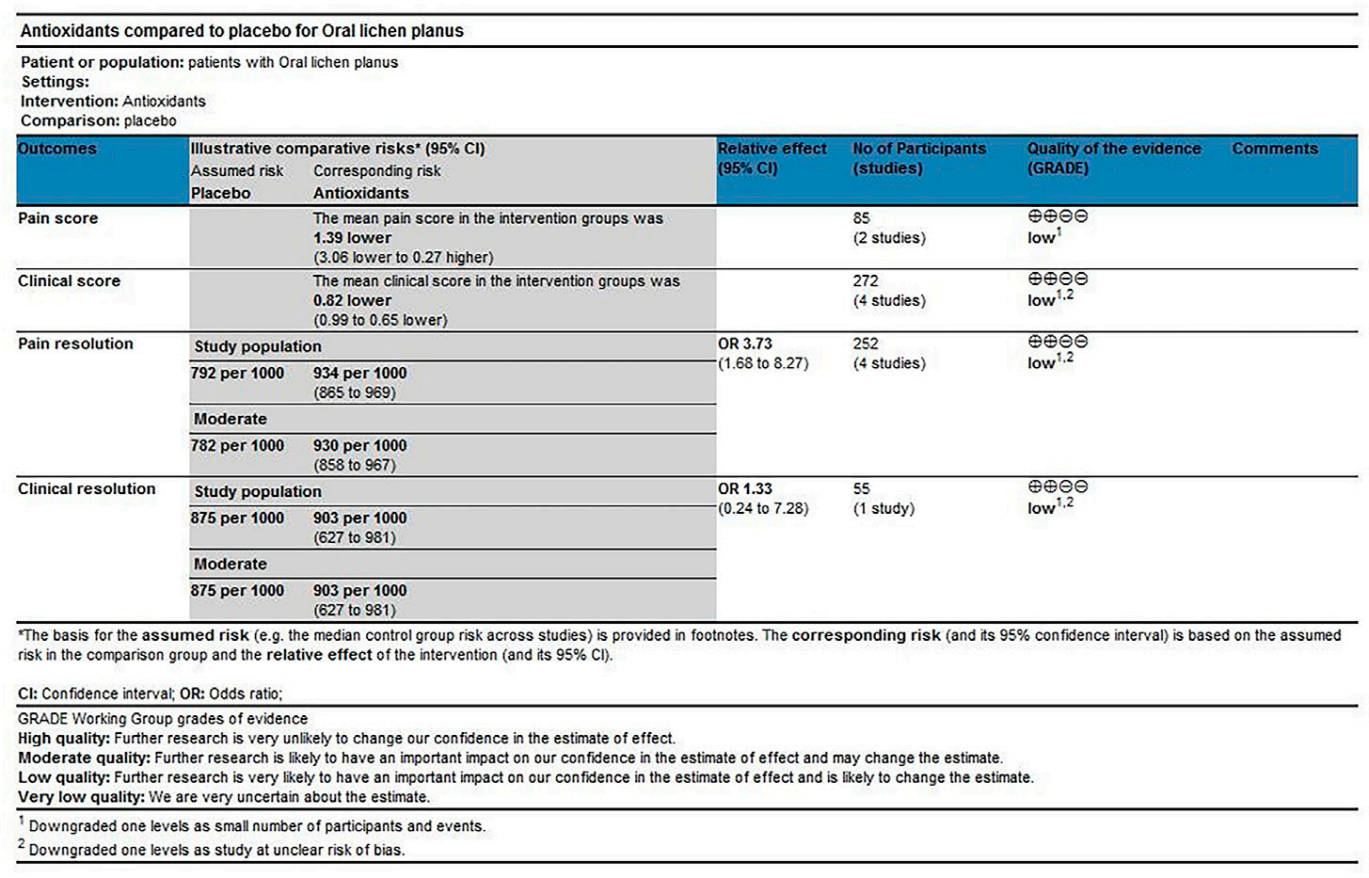
Antioxidants compared with placebo for treating oral lichen planus.

**FIGURE 9:**
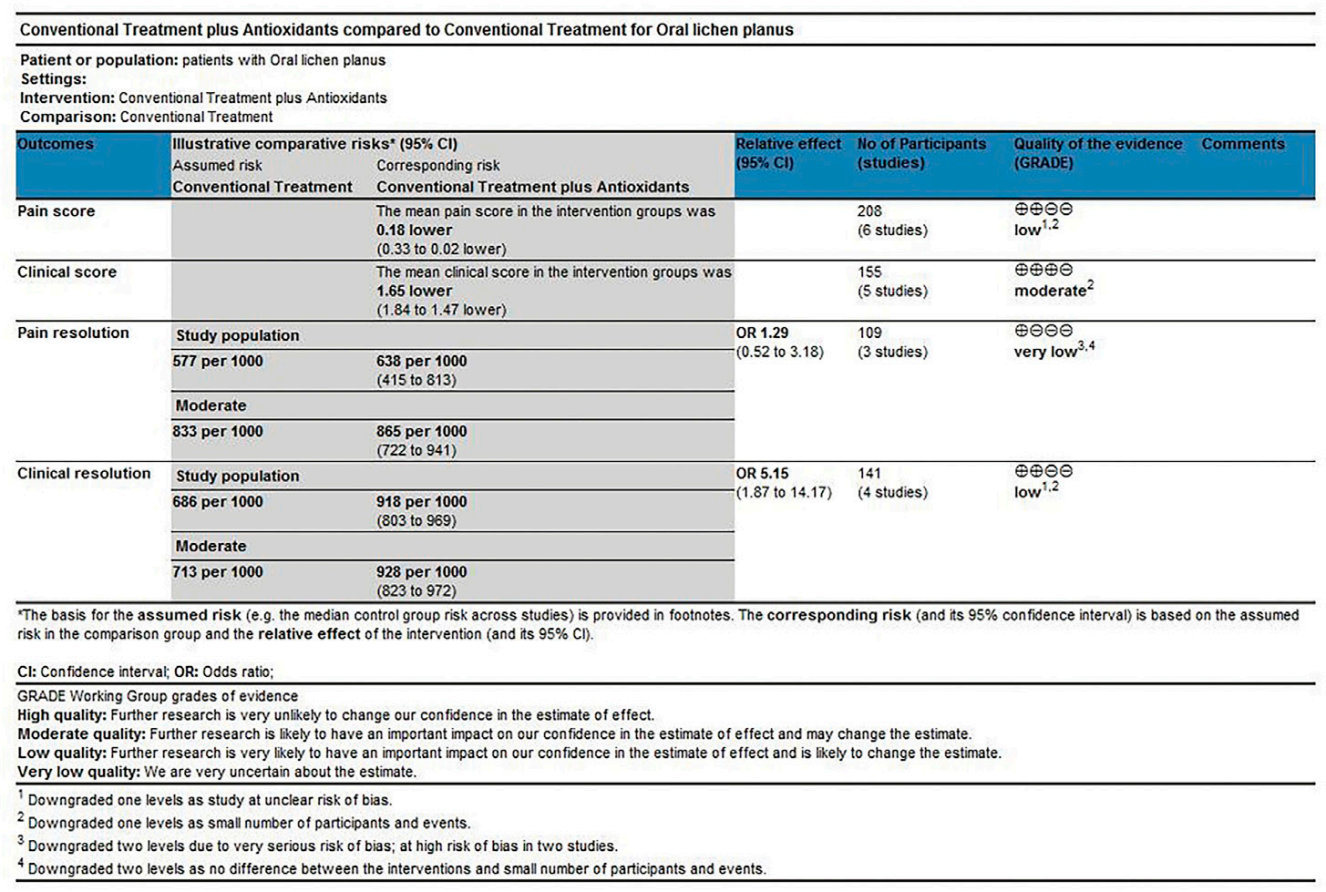
Conventional treatment plus antioxidants compared with conventional treatment for treating oral lichen planus.

The scoring method was used to evaluate the grade of evidence. Two different evaluations were performed: 1) overall evaluation for the effects of antioxidants treatment versus placebo treatment (Figure 8), and 2) overall evaluation for the effects of conventional treatment versus conventional treatment plus antioxidants treatment (Figure 9). The overall analysis showed that the quality of evidence was “very low” for pain resolution rate; the quality of evidence of other endpoints ranged from “low” to “moderate.” The flaws in risk of bias and small sample sizes were directly associated with the downgrade of evidence levels.

## Discussion

The findings of this systematic review showed that antioxidant treatment could reduce the pain and clinical scores and improve the pain and clinical resolution rates of patients with OLP. The meta-analysis of adverse effects showed that the differences in the antioxidants group versus placebo group and the conventional treatment group versus conventional treatment plus antioxidants group were not statistically significant. These findings demonstrated that antioxidants were safe and effective for treating OLP, and antioxidant treatment could be used as a beneficial treatment for patients with OLP. Most endpoints in this meta-analysis showed relatively high heterogeneity. At present, oral lichen planus faces many challenges in terms of etiology, pathogenesis, clinical diagnosis, and treatment. van der Meij et al. (1999) have shown that histopathological diagnoses by observers were subjective and non-reproducible, based on the criteria of the 1978 World Health Organization (WHO) Collaborating Center for Oral Precancerous Lesions, and this criterion did not rule out oral epithelial dysplasia, suggested uncertainty about the value of each patient diagnosed with OLP, who may not be representative of entities with the same disease. Inappropriate diagnosis affected treatment and prognosis, which may be an important reason for heterogeneity in most endpoints. In addition, differences in the type and severity of OLP, individual characteristics (age, sex, diet, smoking, etc.) may affect the effectiveness of antioxidant therapy, leaded to heterogeneity. Sensitivity analyses of pain scores suggested that the study by Shetty et al. (2016) may be another source of heterogeneity, because the baseline of this study was unbalanced. The subgroup analysis showed that the clinical score in patients treated using antioxidants decreased significantly. Further sensitivity analysis by excluding the studies one by one showed that the result was not changed substantially, indicating that the result was robust. Heterogeneity in clinical resolution may also be related to the different measurement methods used in the studies. The quality of evidence was evaluated according to the GRADE scoring criteria, the level of evidence was low quality for the comparison between antioxidant treatment and placebo treatment and ranged from very low quality to moderate quality for the comparison between conventional treatment and conventional treatment plus antioxidants. The recommendation strength of evidence was conditional in this study. Not all the trials used allocation concealment or blinding, and the overall quality of the evidence was restricted by poor study methods and small sample sizes, which led to the downgrade of evidence.

Previous studies demonstrated that the inflammatory infiltration of T cells and cytokines in patients with OLP could stimulate the generation of ROS, while the toxic levels of ROS could upregulate the expression of the intercellular adhesion molecule (ICAM)-1 and consequently damage endothelial cells, which in turn promoted the recruitment of T lymphocytes at the site of inflammatory infiltration, leading to a reciprocal effect. In addition, free radicals could activate nuclear factor-κB, which regulated the expression of inflammatory factors TNF-α and IL-2 and transcribed MHC-I and IL-2 receptor genes, and consequently played important roles in the development and progression of OLP. TNF-α could also induce the formation of hydrogen peroxide (H_2_O_2_) and superoxide anion (O_2_^−^) in epidermal keratinocytes. All these findings demonstrated that elevated ROS could enhance inflammatory responses through immune mechanisms, consequently inducing the occurrence of OLP (Anshumalee et al., 2007; Aly and Shahin, 2010). In addition, the elevation of ROS could induce DNA damage, protein oxidation, and lipid peroxidation, which could jointly exert the effects with cellular membrane damage and lack of repair of cells to induce the malignant transformation of OLP.

A systematic review performed by Jia et al. reported that the level of oxidative stress increased whereas the anti-oxidation level decreased in patients with OLP, demonstrating the important role of oxidative stress in OLP occurrence (Wang et al., 2021). Whether oxidative stress is the cause or result of OLP is unclear. However, the fact is that an imbalance exists between pro-oxidation substances and anti-oxidation systems in patients with OLP, and the relationship between oxidative stress and OLP has already been well established. Therefore, antioxidants can neutralize the adverse effects of oxidative stress to avoid or eliminate oxidative stress–related diseases. Antioxidants restore the impairment affected by free radicals by inhibiting the creation of new radicals, or catching the free radicals to evade chain reaction. We speculated that antioxidants could reduce the interaction between free radicals and inflammatory factors in OLP patients through the above-mentioned process, reduce the production of ROS, and consequently reduce and/or restore cell damage or DNA damage, improve the clinical manifestations. Whether the inflammatory reaction is reduced may be verified by the study of Idrees et al. (2021b) The study is the first to use artificial intelligence to create a machine-learning artificial neural network to identify and quantify monocytes cells and granulocytes within inflammatory infiltration in digitized hematoxylin and eosin microscopic slides. Antioxidants include endogenous and exogenous antioxidants, and endogenous antioxidants include enzymatic and nonenzymatic antioxidants. Endogenous enzymatic antioxidants consist of glutathione peroxidase, superoxide dismutase, and catalase, while nonenzymatic antioxidants consist of nonenzymatic compounds, such as glutathione and proteins, and low–molecular weight scavengers, such as uric acid, coenzyme Q, and lipoic acid. Exogenous antioxidants mainly include carotenoids, vitamin A, C, and E, phenols, resveratrol, and other compounds (Pisoschi and Pop, 2015). The studies included in this systematic review provided information on clinical studies on different antioxidants (Supplementary Material S3). The beneficial effects of these antioxidants in treating OLP also indirectly demonstrated the role of oxidative stress in the pathogenesis of OLP.

The findings of this systematic review on OLP demonstrated that antioxidants could reduce the pain and clinical scores of OLP, and improve the pain and lesion conditions in patients with OLP without increasing adverse effects, indicating that antioxidants could be a beneficial treatment for OLP. However, this meta-analysis had several limitations. First, only relatively few studies were included in the subgroup analysis in this study, and hence more clinical studies are needed to evaluate the endpoints. But more research in the future must be based on accurate diagnosis. To overcome this challenge, the American Academy of Oral and Maxillofacial Pathology (AAOMP) proposed a new set of diagnostic criteria (Cheng et al., 2016) in 2016 by modifying the existing WHO revised criteria, and Idrees et al. (2021c) demonstrated the reliability of using AAOMP criteria to diagnose OLP, which resulted in a more homogeneous population of OLP patients. It is recommended that future researchers follow this criterion for diagnosing OLP, which can help improve the effectiveness of clinical and basic research to study OLP in the future. Second, the types and doses of antioxidants were different among the studies, and it was difficult to evaluate, compare, and analyze the results. In future studies, multiple levels of specific antioxidant doses are needed to assess the optimal effect of antioxidant therapy. Finally, the sample sizes of the included studies were relatively small, and thus the power of investigating the effects of different treatments could be insufficient. It is necessary to expand the study of sample size in the future.

## Conclusion

The findings of this study demonstrated that the treatment using antioxidants could be a potentially effective method for patients with OLP and is worth promoting in clinical practice. However, the sample sizes of previous studies were relatively small. Hence, more randomized controlled trials with larger sample sizes and higher qualities are needed to comprehensively evaluate the clinical efficacy and safety performances of antioxidants in treating patients with OLP.

## Data Availability

The original contributions presented in the study are included in the article/Supplementary Material, further inquiries can be directed to the corresponding authors.
