# Supplementary material for: Antioxidant therapy for patients with oral lichen planus: A systematic review and meta-analysis
Source: Front Pharmacol. 2022 Nov 10;13:1030893. doi: 10.3389/fphar.2022.1030893 (PMC9684670; doi:10.3389/fphar.2022.1030893)
Supplement: Supplementary file 1 [file Table1.DOCX]

Appendix A. (Supplementary data):

Search strategy on each electronic database.

| Database | Search format |
| --- | --- |
| PubMed | ("lichen planus, oral"[MeSH Terms] OR "oral lichen planus"[Title/Abstract]) AND ("Antioxidants"[MeSH Terms] OR "Lycopene"[MeSH Terms] OR "ascorbic acid"[MeSH Terms] OR "Tea"[MeSH Terms] OR "Resveratrol"[MeSH Terms] OR "Selenium"[MeSH Terms] OR "anthocyanins"[MeSH Terms] OR "tocopherols"[MeSH Terms] OR "vitamin a"[MeSH Terms] OR "vitamin e"[MeSH Terms] OR "Curcumin"[MeSH Terms] OR "carotenoids"[MeSH Terms] OR "flavonoids"[MeSH Terms] OR "vitamins"[MeSH Terms] OR ("Anti-Oxidants"[Title/Abstract] OR "Anti-Oxidants"[Title/Abstract] OR "Antioxidant"[Title/Abstract] OR "Anti-Oxidant"[Title/Abstract] OR "Anti-Oxidant"[Title/Abstract] OR "endogenous antioxidants"[Title/Abstract] OR "antioxidants endogenous"[Title/Abstract] OR "endogenous antioxidant"[Title/Abstract] OR "antioxidant endogenous"[Title/Abstract] OR "antioxidant activity"[Title/Abstract] OR "activity antioxidant"[Title/Abstract] OR "antioxidant effect"[Title/Abstract] OR "anti oxidant effect"[Title/Abstract] OR "anti oxidant effect"[Title/Abstract] OR "anti oxidant effects"[Title/Abstract] OR "anti oxidant effects"[Title/Abstract] OR "antioxidant effects"[Title/Abstract]) OR ((((((((("LYC-O-MATO"[Title/Abstract] OR "LYC-O-MATO"[Title/Abstract] OR "LYCOMATO"[Title/Abstract] OR "All-trans-Lycopene"[Title/Abstract] OR "All-trans-Lycopene"[Title/Abstract] OR ("lycopen"[All Fields] OR "Lycopene"[MeSH Terms] OR "Lycopene"[All Fields] OR "lycopenes"[All Fields])) AND ("7-cis"[All Fields] AND "7-cis"[All Fields] AND "9-cis"[All Fields] AND "9-cis"[All Fields])) AND "isomer"[Title/Abstract]) OR "Pro-Lycopene"[Title/Abstract] OR "Pro-Lycopene"[Title/Abstract] OR "Prolycopene"[Title/Abstract] OR ("lycopen"[All Fields] OR "Lycopene"[MeSH Terms] OR "Lycopene"[All Fields] OR "lycopenes"[All Fields])) AND "cis"[All Fields]) AND "isomer"[Title/Abstract]) OR ("lycopen"[All Fields] OR "Lycopene"[MeSH Terms] OR "Lycopene"[All Fields] OR "lycopenes"[All Fields])) AND "13-cis"[All Fields]) AND "isomer"[Title/Abstract]) OR ("acid ascorbic"[Title/Abstract] OR "l ascorbic acid"[Title/Abstract] OR "acid l ascorbic"[Title/Abstract] OR "l ascorbic acid"[Title/Abstract] OR "vitamin c"[Title/Abstract] OR "Hybrin"[Title/Abstract] OR "sodium ascorbate"[Title/Abstract] OR "ascorbate sodium"[Title/Abstract] OR (("Ascorbate"[All Fields] OR "ascorbates"[All Fields] OR "Ascorbic"[All Fields]) AND "acid monosodium salt"[Title/Abstract]) OR "ferrous ascorbate"[Title/Abstract] OR "ascorbate ferrous"[Title/Abstract] OR "magnesium ascorbate"[Title/Abstract] OR "ascorbate magnesium"[Title/Abstract] OR (("Magnesium"[MeSH Terms] OR "Magnesium"[All Fields] OR "magnesium s"[All Fields] OR "magnesiums"[All Fields]) AND "di-L-Ascorbate"[Title/Abstract]) OR (("Magnesium"[MeSH Terms] OR "Magnesium"[All Fields] OR "magnesium s"[All Fields] OR "magnesiums"[All Fields]) AND "di-L-Ascorbate"[Title/Abstract]) OR ("di-L-Ascorbate"[All Fields] AND "Magnesium"[Title/Abstract]) OR (("Magnesium"[MeSH Terms] OR "Magnesium"[All Fields] OR "magnesium s"[All Fields] OR "magnesiums"[All Fields]) AND "Ascorbicum"[Title/Abstract])) OR ("black tea"[Title/Abstract] OR "black teas"[Title/Abstract] OR "tea black"[Title/Abstract] OR "teas black"[Title/Abstract] OR "green tea"[Title/Abstract] OR "green teas"[Title/Abstract] OR "tea green"[Title/Abstract] OR "teas green"[Title/Abstract]) OR ("3 5 4 trihydroxystilbene"[Title/Abstract] OR "3 4 5 trihydroxystilbene"[Title/Abstract] OR "3 4 5 stilbenetriol"[Title/Abstract] OR "trans-Resveratrol-3-O-sulfate"[Title/Abstract] OR "trans-Resveratrol-3-O-sulfate"[Title/Abstract] OR "SRT-501"[Title/Abstract] OR "SRT501"[Title/Abstract] OR "SRT-501"[Title/Abstract] OR "cis-Resveratrol"[Title/Abstract] OR "cis-Resveratrol"[Title/Abstract] OR "trans-Resveratrol"[Title/Abstract] OR "trans-Resveratrol"[Title/Abstract] OR "Resveratrol-3-sulfate"[Title/Abstract] OR "Resveratrol-3-sulfate"[Title/Abstract]) OR ("Selenium-80"[Title/Abstract] OR "Selenium-80"[Title/Abstract]) OR ("Anthocyanin"[Title/Abstract] OR "Leucoanthocyanidins"[Title/Abstract] OR "Anthocyanidins"[Title/Abstract] OR "Anthocyanidin"[Title/Abstract]) OR ((("Tocopherol"[Title/Abstract] OR ("Uno"[All Fields] AND "Vit"[Title/Abstract]) OR ("Vita-Plus"[All Fields] AND "E"[Title/Abstract]) OR (("Vita"[All Fields] AND "Plus"[All Fields]) AND "E"[Title/Abstract]) OR ("VitaPlus"[All Fields] AND "E"[Title/Abstract]) OR (("vitamin s"[All Fields] OR "vitamine"[All Fields] OR "vitamines"[All Fields] OR "vitamins"[Pharmacological Action] OR "vitamins"[MeSH Terms] OR "vitamins"[All Fields] OR "Vitamin"[All Fields]) AND "e al"[Title/Abstract]) OR (("vitamin e"[MeSH Terms] OR "vitamin e"[All Fields]) AND "Natur"[Title/Abstract]) OR (("vitamin e"[MeSH Terms] OR "vitamin e"[All Fields]) AND "Sanum"[Title/Abstract]) OR "vitamin e suspension"[Title/Abstract] OR (("vitamin e"[MeSH Terms] OR "vitamin e"[All Fields]) AND "Dragees"[Title/Abstract]) OR (("vitamin e"[MeSH Terms] OR "vitamin e"[All Fields]) AND "Dragees"[Title/Abstract]) OR (("vitamin s"[All Fields] OR "vitamine"[All Fields] OR "vitamines"[All Fields] OR "vitamins"[Pharmacological Action] OR "vitamins"[MeSH Terms] OR "vitamins"[All Fields] OR "Vitamin"[All Fields]) AND "e gnr"[Title/Abstract]) OR "Vitazell"[Title/Abstract] OR "e vitamin e"[Title/Abstract] OR (("vitamin s"[All Fields] OR "vitamine"[All Fields] OR "vitamines"[All Fields] OR "vitamins"[Pharmacological Action] OR "vitamins"[MeSH Terms] OR "vitamins"[All Fields] OR "Vitamin"[All Fields]) AND "E-mp"[Title/Abstract]) OR (("vitamin s"[All Fields] OR "vitamine"[All Fields] OR "vitamines"[All Fields] OR "vitamins"[Pharmacological Action] OR "vitamins"[MeSH Terms] OR "vitamins"[All Fields] OR "Vitamin"[All Fields]) AND "E-mp"[Title/Abstract]) OR (("vitamin s"[All Fields] OR "vitamine"[All Fields] OR "vitamines"[All Fields] OR "vitamins"[Pharmacological Action] OR "vitamins"[MeSH Terms] OR "vitamins"[All Fields] OR "Vitamin"[All Fields]) AND "Emp"[Title/Abstract]) OR "E-ferol"[Title/Abstract] OR "E-Mulsin"[Title/Abstract] OR "E-Mulsin"[Title/Abstract] OR "E-Vicotrat"[Title/Abstract] OR "E-Vicotrat"[Title/Abstract] OR ("Elex"[All Fields] AND "Verla"[Title/Abstract]) OR "Evion"[Title/Abstract] OR "Ephynal"[Title/Abstract] OR ("Hydrovit"[All Fields] AND "E"[Title/Abstract]) OR ("Malton"[All Fields] AND "E"[Title/Abstract]) OR ("Puncto"[All Fields] AND "E"[Title/Abstract]) OR ("Snow-E"[All Fields] AND "muscle energy"[Title/Abstract])) AND "Feritility"[Title/Abstract]) OR ("Dal"[All Fields] AND "E"[Title/Abstract]) OR "aquasol e"[Title/Abstract] OR ("Auxina"[All Fields] AND "E"[Title/Abstract]) OR "bio e"[Title/Abstract] OR "Biosan"[Title/Abstract] OR "Lasar"[Title/Abstract] OR "Davitamon"[Title/Abstract] OR "Spondyvit"[Title/Abstract] OR ("Vit"[All Fields] AND "e hydrosol"[Title/Abstract]) OR (("vitamin e"[MeSH Terms] OR "vitamin e"[All Fields] OR "vit e"[All Fields]) AND "Stada"[Title/Abstract]) OR "Vita-E"[Title/Abstract] OR "Vita-E"[Title/Abstract] OR "VitaE"[Title/Abstract] OR (("tocopherols"[MeSH Terms] OR "tocopherols"[All Fields] OR "Tocopherol"[All Fields] OR "tocopheryl"[All Fields]) AND "Bayer"[Title/Abstract]) OR "unique e"[Title/Abstract]) OR (((((("aquasol a"[Title/Abstract] OR "Retinol"[Title/Abstract] OR ("3"[All Fields] AND "7 dimethyl 9"[All Fields])) AND ("2"[All Fields] AND "6"[All Fields] AND "6-trimethyl-1-cyclohexen-1-yl"[All Fields])) AND ("2"[All Fields] AND "4"[All Fields] AND "6"[All Fields] AND "8-nonatetraen-1-ol"[All Fields])) AND "all-E"[All Fields]) AND "isomer"[Title/Abstract]) OR "All-Trans-Retinol"[Title/Abstract] OR "All-Trans-Retinol"[Title/Abstract] OR "vitamin a1"[Title/Abstract] OR "11-cis-Retinol"[Title/Abstract]) OR ("turmeric yellow"[Title/Abstract] OR "yellow turmeric"[Title/Abstract] OR "curcumin phytosome"[Title/Abstract] OR (("phytosomal"[All Fields] OR "Phytosome"[All Fields] OR "phytosomes"[All Fields]) AND "Curcumin"[Title/Abstract]) OR "Diferuloylmethane"[Title/Abstract]) OR ("Carotenoid"[Title/Abstract] OR "Tetraterpenes"[Title/Abstract] OR ("Tetraterpene"[All Fields] AND "Derivatives"[Title/Abstract]) OR (("analogs and derivatives"[MeSH Subheading] OR ("analogs"[All Fields] AND "Derivatives"[All Fields]) OR "analogs and derivatives"[All Fields] OR "Derivatives"[All Fields] OR "derivable"[All Fields] OR "derivant"[All Fields] OR "derivants"[All Fields] OR "derivate"[All Fields] OR "derivated"[All Fields] OR "derivates"[All Fields] OR "derivation"[All Fields] OR "derivations"[All Fields] OR "derivative"[All Fields] OR "derive"[All Fields] OR "derived"[All Fields] OR "derives"[All Fields] OR "deriving"[All Fields]) AND "Tetraterpene"[Title/Abstract]) OR "Carotenes"[Title/Abstract] OR "Carotene"[Title/Abstract]) OR ((("2"[All Fields] AND ("Phenyl"[All Fields] OR "phenylated"[All Fields] OR "phenylation"[All Fields] OR "phenylic"[All Fields] OR "phenyls"[All Fields])) AND "Chromenes"[Title/Abstract]) OR "2-Phenyl-Benzopyran"[Title/Abstract] OR "2-Phenyl-Benzopyran"[Title/Abstract] OR ("2"[All Fields] AND "phenyl benzopyrans"[Title/Abstract]) OR "2-Phenyl-Chromene"[Title/Abstract] OR "2-Phenyl-Chromene"[Title/Abstract] OR "Flavonoid"[Title/Abstract] OR "Bioflavonoids"[Title/Abstract] OR "Bioflavonoid"[Title/Abstract]) OR "Vitamin"[Title/Abstract]) AND ("randomized controlled trial"[Publication Type] OR "controlled clinical trial"[Publication Type] OR "Randomized"[Title/Abstract] OR "placebo"[Title/Abstract] OR "randomly"[Title/Abstract] OR "Trial"[Title] OR "randomized controlled trials as topic"[MeSH Terms] OR ("Controlled"[All Fields] AND "clinical trials as topic"[MeSH Terms]) OR "random allocation"[Title/Abstract] OR "double-blind"[Title/Abstract] OR "single-blind"[Title/Abstract]) |
| Web of Science | (((((((((((((((TS=(Antioxidants or “Activity, Antioxidant” or “Antioxidant Activity” or Anti-Oxidants or Antioxidant or “Anti Oxidants” or “Anti Oxidant” or Anti-Oxidant or “Endogenous Antioxidant” or “Antioxidant, Endogenous” or “Endogenous Antioxidants” or “Antioxidants, Endogenous” or “Anti Oxidant Effect” or “Antioxidant Effect” or “Anti-Oxidant Effect” or “Anti Oxidant Effects” or “Antioxidant Effects” or “Anti-Oxidant Effects”)) OR TS=(Lycopene or “All-trans-Lycopene” or “All trans Lycopene” or “LYC O MATO” or LYCOMATO or LYC-O-MATO or Pro-Lycopene or Prolycopene or “Pro Lycopene”)) OR TS=(“Ascorbic acid” or “Acid, L-Ascorbic” or “L-Ascorbic Acid” or “L Ascorbic Acid” or “Acid, Ascorbic” or “Vitamin C” or Magnorbin or “Ascorbate, Sodium” or “Ascorbic Acid, Monosodium Salt” or “Sodium Ascorbate” or Hybrin or “Magnesium di-L-Ascorbate” or “Magnesium Ascorbicum” or “Ascorbate, Magnesium” or “Magnesium di L Ascorbate” or “di-L-Ascorbate, Magnesium” or “Magnesium Ascorbate” or “Ferrous Ascorbate” or “Ascorbate, Ferrous”)) OR TS=(Tea or “Tea, Black” or “Black Tea” or “Teas, Black” or “Black Teas” or “Green Tea” or “Teas, Green” or “Green Teas” or “Tea, Green”)) OR TS=(Resveratrol or SRT-501 or “SRT 501” or SRT501 or “trans Resveratrol 3 O sulfate” or “trans Resveratrol” or trans-Resveratrol or “cis Resveratrol” or cis-Resveratrol or “Resveratrol 3 sulfate”)) OR TS=(Selenium or “Selenium 80” or Selenium-80)) OR TS=(Anthocyanins or Anthocyanidin or Anthocyanidins or Leucoanthocyanidins or Anthocyanin)) OR TS=(Tocopherols or Bioweyxin or E-Vitamin-Ratiopharm or “Snow-E Muscle, Energy & Feritility” or “Unique E” or Tocopa or “Bio E” or Detulin or “Togasan Vitamin E” or “Vitamin E, Togasan” or “Hydrovit E” or “Micorvit E” or Vitazell or Tocopharm or “Vitamine E GNR” or “E Mulsin” or E-Mulsin or “Aquasol E” or “Vitamin E Natur” or “Vitamin-E EVI-MIRALE” or “Vitamin E EVI MIRALE” or “VitaminE EVIMIRALE” or “Vitamin E Sanum” or “Vit. E Stada” or “Puncto E” or “Equivit E” or Uno-Vit or UnoVit or “Uno Vit” or “VitaPlus E” or “Vita Plus E” or “Vita-Plus E” or Eusovit or “Dal E” or Dal-E or Biopto-E or E-ferol or Vibolex or E-Vicotrat or E Vicotrat or Dermorelle or Tocolion or “Vitamin-E Dragees” or “Vitamin E Dragees” or “Auxina E” or “Richtavit E” or Eplonat or “Vita E” or Vita-E or VitaE or Lasar or Biosan or Davitamon or “Sanavitan S” or Tocopherol or “Vitamin E Suspension” or Ephynal or “Vit E hydrosol” or “Malton E” or “Elex Verla” or “Antioxidans E-Hevert” or “EUNOVA Vitamin E” or “Vitamin E, Mowivit” or “Mowivit Vitamin E” or Ecoro or Spondyvit or Embial or Evion or “Vitamin E AL” or Abortosan or “Vitamin E, Vitagutt” or “Vitagutt Vitamin E” or “Tocopherol Bayer” or “Vitamin E-mp” or “E Vitamin E” or “Vitamin Emp” or “Vitamin E mp” or Tocovital)) OR TS=(“Vitamin A” or Retinol or “All Trans Retinol” or “Vitamin A1” or All-Trans-Retinol or “Aquasol A”)) OR TS=(“Vitamin E”)) OR TS=(Curcumin or “Phytosome, Curcumin” or “Yellow, Turmeric” or “Turmeric Yellow” or Diferuloylmethane or “Curcumin Phytosome” or Mervia)) OR TS=(Flavonoids or “2 Phenyl Benzopyrans” or “2 Phenyl Chromene” or Flavonoid or “2 Phenyl Chromenes” or “2 Phenyl Benzopyran” or Bioflavonoid or Bioflavonoids)) OR TS=(Carotenoids or “Tetraterpene Derivatives” or Tetraterpenes or “Derivatives, Tetraterpene” or Carotenoid or Carotenes or Carotene)) OR TS=(Vitamins or Vitamin)) AND TS=(“oral lichen planus” or “lichen planus, oral”)) AND TS=("randomized controlled trial" or "controlled clinical trial" or Randomized or placebo or randomly or Trial or Controlled or "clinical trials " or "random allocation" or "double blind" or "single blind" or double-blind or single-blind) |
| Cochrane | #1 MeSH descriptor: [Antioxidants] explode all trees 5166  #2 MeSH descriptor: [Lycopene] explode all trees 271  #3 MeSH descriptor: [Ascorbic Acid] explode all trees 2350  #4 MeSH descriptor: [Tea] explode all trees 542  #5 MeSH descriptor: [Resveratrol] explode all trees 322  #6 MeSH descriptor: [Anthocyanins] explode all trees 159  #7 MeSH descriptor: [Selenium] explode all trees 756  #8 MeSH descriptor: [Tocopherols] explode all trees 771  #9 MeSH descriptor: [Vitamin A] explode all trees 2143  #10 MeSH descriptor: [Vitamin E] explode all trees 2628  #11 MeSH descriptor: [Curcumin] explode all trees 502  #12 MeSH descriptor: [Flavonoids] explode all trees 2959  #13 MeSH descriptor: [Carotenoids] explode all trees 3833  #14 MeSH descriptor: [Vitamins] explode all trees 5129  #15 #1 or #2 or #3 or #4 or #5 or #6 or #7 or #8 or #9 or #10 or #11 or #12 or #13 or #14 18594  #16 (Activity, Antioxidant or Antioxidant Activity or Anti-Oxidants or Antioxidant or Anti Oxidants or Anti Oxidant or Anti-Oxidant or Endogenous Antioxidant or Antioxidant, Endogenous or Endogenous Antioxidants or Antioxidants, Endogenous or Anti Oxidant Effect or Antioxidant Effect or Anti-Oxidant Effect or Anti Oxidant Effects or Antioxidant Effects or Anti-Oxidant Effects):ti,ab,kw (Word variations have been searched) 17179  #17 (All-trans-Lycopene or All trans Lycopene or LYC O MATO or LYCOMATO or LYC-O-MATO or Pro-Lycopene or Prolycopene or Pro Lycopene):ti,ab,kw (Word variations have been searched) 62  #18 (Acid, L-Ascorbic or L-Ascorbic Acid or L Ascorbic Acid or Acid, Ascorbic or Vitamin C or Magnorbin or Ascorbate, Sodium or Ascorbic Acid, Monosodium Salt or Sodium Ascorbate or Hybrin or Magnesium di-L-Ascorbate or Magnesium Ascorbicum or Ascorbate, Magnesium or Magnesium di L Ascorbate or di-L-Ascorbate, Magnesium or Magnesium Ascorbate or Ferrous Ascorbate or Ascorbate, Ferrous):ti,ab,kw (Word variations have been searched) 9994  #19 (Tea, Black or Black Tea or Teas, Black or Black Teas or Green Tea or Teas, Green or Green Teas or Tea, Green):ti,ab,kw (Word variations have been searched) 1499  #20 (SRT-501 or SRT 501 or SRT501 or trans Resveratrol 3 O sulfate or trans Resveratrol or trans-Resveratrol or cis Resveratrol or cis-Resveratrol or Resveratrol 3 sulfate):ti,ab,kw (Word variations have been searched) 94  #21 (Selenium 80 or Selenium-80):ti,ab,kw (Word variations have been searched) 250  #22 (Anthocyanidin or Anthocyanidins or Leucoanthocyanidins or Anthocyanin):ti,ab,kw (Word variations have been searched) 571  #23 (Bioweyxin or E-Vitamin-Ratiopharm or Snow-E Muscle, Energy & Feritility or Unique E or Tocopa or Bio E or Detulin or Togasan Vitamin E or Vitamin E, Togasan or Hydrovit E or Micorvit E or Vitazell or Tocopharm or Vitamine E GNR or E Mulsin or E-Mulsin or Aquasol E or Vitamin E Natur or Vitamin-E EVI-MIRALE or Vitamin E EVI MIRALE or VitaminE EVIMIRALE or Vitamin E Sanum or Vit. E Stada or Puncto E or Equivit E or Uno-Vit or UnoVit or Uno Vit or VitaPlus E or Vita Plus E or Vita-Plus E or Eusovit or Dal E or Dal-E or Biopto-E or E-ferol or Vibolex or E-Vicotrat or E Vicotrat or Dermorelle or Tocolion or Vitamin-E Dragees or Vitamin E Dragees or Auxina E or Richtavit E or Eplonat or Vita E or Vita-E or VitaE or Lasar or Biosan or Davitamon or Sanavitan S or Tocopherol or Vitamin E Suspension or Ephynal or Vit E hydrosol or Malton E or Elex Verla or Antioxidans E-Hevert or EUNOVA Vitamin E or Vitamin E, Mowivit or Mowivit Vitamin E or Ecoro or Spondyvit or Embial or Evion or Vitamin E AL or Abortosan or Vitamin E, Vitagutt or Vitagutt Vitamin E or Tocopherol Bayer or Vitamin E-mp or E Vitamin E or Vitamin Emp or Vitamin E mp or Tocovital):ti,ab,kw (Word variations have been searched) 13422  #24 (Retinol or All Trans Retinol or Vitamin A1 or All-Trans-Retinol or Aquasol A):ti,ab,kw (Word variations have been searched) 2322  #25 (Phytosome, Curcumin or Yellow, Turmeric or Turmeric Yellow or Diferuloylmethane or Curcumin Phytosome or Mervia):ti,ab,kw (Word variations have been searched) 64  #26 (2 Phenyl Benzopyrans or 2 Phenyl Chromene or Flavonoid or 2 Phenyl Chromenes or 2 Phenyl Benzopyran or Bioflavonoid or Bioflavonoids):ti,ab,kw (Word variations have been searched) 1696  #27 (Tetraterpene Derivatives or Tetraterpenes or Derivatives, Tetraterpene or Carotenoid or Carotenes or Carotene):ti,ab,kw (Word variations have been searched) 2933  #28 (Vitamin):ti,ab,kw (Word variations have been searched) 33841  #29 #16 or #17 or #18 or #19 or #20 or #21 or #22 or #23 or #24 or #25 or #26 or #27 or #28 57077  #30 #15 or #29 60847  #31 MeSH descriptor: [Lichen Planus, Oral] explode all trees 189  #32 (oral lichen planus):ti,ab,kw (Word variations have been searched) 488  #33 #31 or #32 488  #34 #30 and #33 60 |
| Embase | ('oral lichen planus'/exp/mj OR 'lichen planus'/exp/mj) AND ('antioxidant'/exp OR 'tea'/exp OR 'anthocyanin'/exp OR 'tocopherol'/exp OR 'retinol'/exp OR 'ascorbic acid'/exp OR 'alpha tocopherol'/exp OR 'carotenoid'/exp OR antioxidant$ OR vitamin$ OR 'selenium'/de OR carnitine$ OR carotenoid$ OR lycopene$ OR multivitamin$ OR betacarotene$ OR 'beta carotene$' OR 'ascorbic acid'/de OR 'alpha tocopherol$' OR flavonoid$ OR 'melatonin'/de OR 'curcumin'/de OR 'resveratrol'/de) AND ('randomized controlled trial'/exp/mj AND topic OR 'controlled clinical trial (topic)'/mj OR randomized OR 'placebo'/mj OR randomly OR 'randomization'/mj OR 'double blind procedure'/mj OR 'single blind procedure'/mj) |
| Google Scholar | “randomized controlled trial” OR “controlled clinical trial” “oral lichen planus” antioxidant |
