# Supplementary material for: Antioxidant therapy for patients with oral lichen planus: A systematic review and meta-analysis
Source: Front Pharmacol. 2022 Nov 10;13:1030893. doi: 10.3389/fphar.2022.1030893 (PMC9684670; doi:10.3389/fphar.2022.1030893)
Supplement: Supplementary file 4 [file Table3.DOCX]

Table b. Antioxidant tested in the included trials

| Study ID | Antioxidant (type: Ex; Vit; Whole) | Dose | Form taken | Regimen | Duration |
| --- | --- | --- | --- | --- | --- |
| Abdeldayem 2020** | Vit: vitamin E | 400 mg/day | Capsules | 1 capsule daily | four weeks |
| Agha-Hosseini 2010 | Ex: purslane extract | 235 mg/day | Capsules | 1 capsule daily | 6 months |
| Agha-Hosseini 2021 | Ex: hyaluronic acid solution formulation | 7 mg hyaluronic acid powder  40 mg/mL triamcinolone acetonide; 0.5ml/cm^2^ | Injection | Not known | 6 months |
| Amirchaghmaghi 2016** | Ex: curcumin | 4000 mg/day of curcumin | Tablets | 8 tablets daily (2×4 tablets) | 4 weeks |
| Bakhshi 2020 | Ex: nanocurcumin gel | 1% Nanocurcumin | Apply | 3 times a day | 4 weeks |
| Chainani-Wu 2007 | Ex: curcuminoids | 2000 mg/day | Capsules | 1 capsule daily | 7 weeks |
| Chainani-Wu 2011** | Ex: curcuminoids | 6000 mg/day | Tablets | 3 tablets daily (3×1 tablets) | 2 weeks |
| Choonhakarn 2008 | Ex: aloe vera gel | 70% aloe vera mucilage | Apply | twice daily | 8 weeks |
| Ghada Nabil 2016 | Ex: green tea | 200 mg/day | tablets | 1 tablet daily | 4 weeks |
| Mostafa 2018 | Ex: gaseous ozone | ozone with an intensity of 60% | ozone generator | twice a week | 4 weeks |
| Nolan 2009 | Ex: hyaluronic acid gel | 0.2% hyaluronic acid | Apply | 4-5 times a day | 4 weeks |
| Saawarn 2011 | Ex: lycopene | 24mg/day | Capsules | 3 capsules daily | 8 weeks |
| Salazar-Sanchez 2010 | Ex: aloe vera gel | 70% aloe vera mucilage  1.2ml/day | Apply | 3 times a day (3×0.4ml) | 12 weeks |
| Sanatkhani 2014 | Whole: cedar honey | 60ml/day | swish and swallow | 3 times a day (3×20ml) | 4 weeks |
| Shetty 2016 | Ex: orabase | 0.2% hyaluronic acid | Apply | 3 times a day | 6 weeks |
| Shoukheba 2016 | Ex: CoQ10 | 90mg/day | Capsules | 3 capsules daily | 12 weeks |
| Veneri 2020 | Ex: ozonized water | double-distilled water to ozone ratio being 2:3  4min/time | oral rinses | twice a week | 12 weeks |

* Types: Extract, mixed or other (e.g. amino-acid derivative) antioxidants; Vitamin; and Whole natural food source.

** Study included in this review used a dose higher than the recommended daily amount.

According to the above findings, antioxidant doses were higher than the recommended daily amount in 3 studies, but there was no significant difference in the incidence of adverse events between the test and control groups. Dose-response effects and time-response effects play an important role in clinical medication. However, due to the variety of antioxidants, there is no perfect study on specific antioxidant dosage at present. In future research, multiple levels of dosage or intervention duration are required to evaluate the dose-response effects or time-response effects and determine the optimal dosage or intervention duration.
