# Supplementary material for: Antioxidant therapy for patients with oral lichen planus: A systematic review and meta-analysis
Source: Front Pharmacol. 2022 Nov 10;13:1030893. doi: 10.3389/fphar.2022.1030893 (PMC9684670; doi:10.3389/fphar.2022.1030893)
Supplement: Supplementary file 6 [file Table6.DOCX]

**Identification of studies via other methods**

**Identification of studies via databases and registers**

Records removed *before screening*:

Duplicate records removed (n =84)

Records marked as ineligible by automation tools (n =0)

Records removed for other reasons (n =0)

Records identified from*:

Databases (n =1150)

PubMed (n =47)

Web of Science (n =89)

Cochrane (n =60)

Embase (n =77)

Google Scholar (n =877)

Records identified from:

Websites (n =0)

Organisations (n =0)

Citation searching (n =4)

**Identification**

Records excluded**

(n = 1026)

Wrong study design (n=567)

Wrong drug (n=362)

Wrong population (n=94)

Unable to source abstract (n=3)

Records screened

(n =1066)

Reports not retrieved

(n =0)

Reports sought for retrieval

(n =0)

Reports sought for retrieval

(n =40)

Reports not retrieved

(n =0)

**Screening**

Reports excluded (n = 0)

Reports excluded:

Nonconformities on control groups (n =15)

Absence of antioxidants (n =9)

Outcome indicators discrepancy (n =1)

Reports assessed for eligibility

(n =4)

Reports assessed for eligibility

(n =40)

Studies included in review

(n =19)

Reports of included studies

(n =17)

**Included**

*Consider, if feasible to do so, reporting the number of records identified from each database or register searched (rather than the total number across all databases/registers).

**If automation tools were used, indicate how many records were excluded by a human and how many were excluded by automation tools.

*From:*  Page MJ, McKenzie JE, Bossuyt PM, Boutron I, Hoffmann TC, Mulrow CD, et al. The PRISMA 2020 statement: an updated guideline for reporting systematic reviews. BMJ 2021;372:n71. doi: 10.1136/bmj.n71. For more information, visit: <http://www.prisma-statement.org/>
